# Signaling pathways that activate hepatic stellate cells during liver fibrosis

**DOI:** 10.3389/fmed.2024.1454980

**Published:** 2024-09-18

**Authors:** Youtian Zhang, Long Ren, Yinting Tian, Xiaohu Guo, Fengxian Wei, Yawu Zhang

**Affiliations:** ^1^The Second Hospital of Lanzhou University, Lanzhou, China; ^2^The Department of General Surgery, The Second Hospital of Lanzhou University, Lanzhou, China; ^3^The Laboratory of Hepatic-Biliary-Pancreatic, The Second Hospital of Lanzhou University, Lanzhou, China

**Keywords:** liver fibrosis, HSCs, activation, signaling pathways, review

## Abstract

Liver fibrosis is a complex process driven by various factors and is a key feature of chronic liver diseases. Its essence is liver tissue remodeling caused by excessive accumulation of collagen and other extracellular matrix. Activation of hepatic stellate cells (HSCs), which are responsible for collagen production, plays a crucial role in promoting the progression of liver fibrosis. Abnormal expression of signaling pathways, such as the TGF-β/Smads pathway, contributes to HSCs activation. Recent studies have shed light on these pathways, providing valuable insights into the development of liver fibrosis. Here, we will review six signaling pathways such as TGF-β/Smads that have been studied more in recent years.

## 1 Introduction

Liver fibrosis is a condition caused by various factors, leading to an excessive buildup of proteins such as collagen in the liver tissue. It is commonly associated with chronic liver diseases and can progress insidiously. Serious disorders like cirrhosis, portal hypertension, and liver failure can be brought on by advanced liver fibrosis and frequently require liver transplantation (1). Regrettably, there are still no effective drugs approved in clinical practice to delay the progression of liver fibrosis, and treatment options are limited to addressing specific causes.

More and more studies have shown a strong correlation between the activation of HSCs and the occurrence and progression of liver fibrosis (2). The essence of liver fibrosis is extracellular matrix (ECM) overproduction which is primarily product by fibroblast myofibroblasts. Myofibroblasts are uncommon in healthy tissues, and HSCs are the primary precursors of myofibroblasts. They originate from mesothelial cells during embryonic development and are located in the subendothelial space of Disse. In a healthy liver, HSCs are inactive. However, when stimulated by profibrotic factors, HSCs become activated and transform into myofibroblast-like cells, playing an active role in tissue repair. Activated HSCs exhibit functions that inactive HSCs do not possess, such as contractility, proliferation, and enhanced ECM synthesis (3). Multiple signaling pathways, play a crucial role in HSCs activation (4). Once activated, HSCs undergo proliferation, enhance contractility, and transform into myofibroblast-like cells that secrete a large amount of ECM, driving liver tissue remodeling and advancing liver fibrosis.

In recent years, a large number of studies have focused on the activation of HSCs in liver fibrosis by TGF-β/Smad, MAPK, PI3K/AKT, Wnt, NF-κB, and AMPK signaling pathways. Therefore, this article places emphasis on the connection between above six signaling pathways and HSCs activation, delving into the mechanism of hepatic stellate cell activation and hepatic fibrosis from a molecular standpoint.

## 2 The independent role of six signaling pathways in HSCs activation and hepatic fibrosis

As mentioned above, six signaling pathways play a very important role in the occurrence and development of liver fibrosis through the activation of HSCs. Next, we will focus on the role of each of the six signaling pathways in liver fibrosis in recent years, especially their respective relationships with HSCs activation. More interestingly, we reviewed how these signals changed during HSCs quiescence, activation, aging or apoptosis.

### 2.1 TGF-β/Smad signal path

**FIGURE 1:**
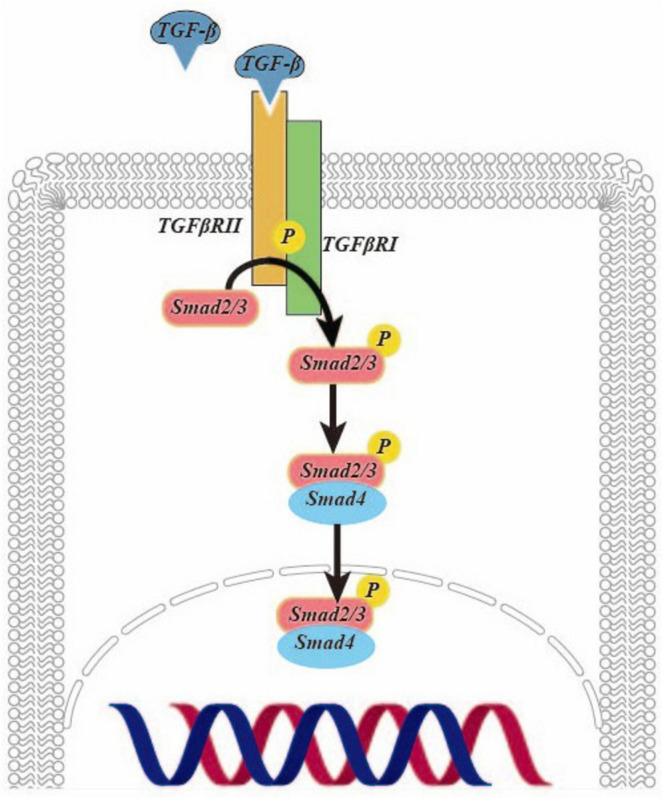
The TGF-β /Smad signaling pathway activates hepatic stellate cells in liver fibrosis.

Transforming growth factor (TGF)-β is a pivotal cytokine with diverse biological functions and a critical role in driving fibrosis. Recent research has underscored the potential of TGF-β as a key target for combatting liver fibrosis (5). It exerts potent effects in activating HSCs (6). Studies have demonstrated that oxoglaucine, when administered as a preconditioning agent, mitigates the TGF-β-induced the upregulation of α-smooth muscle actin (α-SMA) and Collagen Type I (Col I) in Hepa1c1c7 cells. This underscores the promising potential of oxoglaucine as a therapeutic intervention in the context of fibrosis (7). In Figure 1, TGF-β is activated to form a dimer, which binds to and phosphorylates the TGF-β type II receptor on the cell surface, and then interacts with and activates the TGF-β type I receptor. The latter recruits and phosphorylates Smad2/3. This complex then further recruits Smad4, forming the SMAD2/3/4 complex. Finally, this complex is transported to the nucleus to regulate transcription (8).

In the TGF-β/Smad signaling pathway, Smad2/3 is responsible for promoting fibrosis, while Smad7 acts to prevent disease progression (9). Upon TGF-β activation of HSCs, the levels of α-SMA and Col I are significantly elevated compared to the control group. Notably, there is a parallel increase in the Smad3 levels and phosphorylated Smad3. However, the expression of phosphorylated Smad3 is reduced following transfection with the plasmid of left-right determination factor, indicative of the profibrotic effect of Smad3 activation. It is evident that inhibiting its activation can effectively delay the progression of liver fibrosis (9). The TGF-β/Smad signaling pathway activation leads to the localization of the Smad2/3 complex in the nucleus. However, recent studies have shown that when HSCs are co-cultured with adiposederived stem cells or hepatocyte growth factor, the complex is instead distributed throughout the entire cell. Additionally, the study revealed a significant reduction in the levels of α-SMA and Col I expression at both the gene and protein levels compared to activated HSCs. These findings suggest a promising potential for down-regulating this signaling pathway as an effective treatment for liver fibrosis (10). In a groundbreaking study, it was discovered that when human HSCs LX-2 cells were treated with TGF-β and incubated with lactic acid bacteria, there was a remarkable restoration of normal levels of α-SMA, Col I and phosphorylation of Smad2/3 compared to when lactic acid bacteria were absent. Additionally, the expression level of Smad7 increased, suggesting that lactic acid bacteria have the potential to effectively inhibit fibrosis and reduce proinflammatory markers by suppressing the activation of HSCs *in vitro* (11).

Additionally, as a member of the GTPase family, Ras-related protein 31 (Rab31) is the most important factor in the regulation of endocytosis and cell membrane transport. When HSCs are stimulated by TGF-β, Rab31 elimination can inhibit the activation process, leading to p-Smad2’s expression level decreasing. As previously mentioned, the binding of TGF-βRII to TGF-βRI and its subsequent endocytosis are necessary for the TGF-β signaling pathway. When HSCs were stimulated with TGF-β1, in particular, the cell membrane and cytoplasm showed a significant increase in the coexpression of TGF-βRII and EEA-1 proteins. However, Rab31 knockdown reduced the co-expression of both proteins, indicating that Rab31 promotes the endocytosis of the TGF-βRII complex. Rab31’s involvement in activating the TGF-β/Smad signaling pathway can be traced back to the promotion of endocytosis of the TGF-βRII complex, which ultimately leads to the activation of HSCs. The activation of HSCs is regulated by the TGF-β/Smad signaling pathway, as suggested by these findings, which contributes to the progression of liver fibrosis (12).

### 2.2 MAPK signal path

In the world of mammalian biology, one enzyme stands out for its significance: mitogen-activated protein kinase (MAPK). It is worth noting that all eukaryotic cells express MAPK (13). Initially known for its involvement in cell mitosis, MAPK is a protein that binds to serine-threonine. As research has progressed, it has been discovered that MAPK not only plays a crucial role in the growth and differentiation of cells, but also in pathophysiological processes such as inflammation, regulation of lipid metabolism and liver fibrosis (14, 15). MAPK is a family of proteins and currently includes subtypes like ERK, JNK, and p38 (16).

**FIGURE 2:**
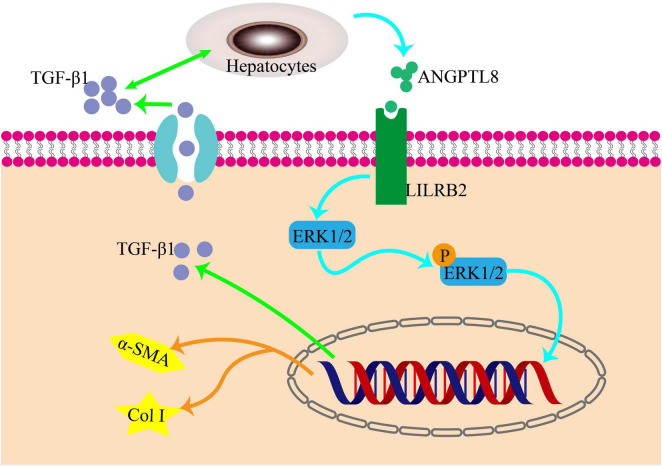
The pro-inflammatory factor ANGPTL8 activates liver stellate cells through the LILRB2/ERK signaling pathway to promote fibrosis. Following a long-term highfat diet, AngPTL8 expression in the liver was increased. The secretion of the inflammatory cytokines ANGPTL8 and TGF-β1 mutually promote each other. ANGPTL8 binds to the LILRB2 receptor on the cell membrane of HSCs, activates HSCs by triggering downstream ERK signals, and subsequently induces the expression of fibrosis factors α-SMA and Col I.

ERK, a crucial member of the MAPK family, plays a significant role in the development of liver fibrosis. The production of type I collagen is controlled by the ERK pathway through the phosphorylation of its downstream molecules, as demonstrated by numerous studies, thereby exacerbating liver fibrosis. Conversely, inhibiting the phosphorylation of the ERK pathway can decrease the expression of Col I (17–19). Recently, a team utilized U0126 to block the phosphorylation of the ERK pathway, which led to a decrease in the expression of paxillin-induced Col I. This study further illustrates the involvement of the ERK pathway in liver fibrosis and highlights the potential of therapeutic drugs that target this pathway (20). When studying the role of ANGPTL8 (also known as lipasin) in the progression of liver fibrosis, it was found that ANGPTL8 is a secreted protein that is highly expressed in the liver during eating and lowly expressed during fasting. It was observed that ANGPTL8 reached the surface of HSCs and bound to its receptor, further activating HSCs activation mediated by the ERK signaling pathway. This eventually leads to liver fibrosis, as shown in Figure 2 (21).

The active compound β-citronellol (β-CIT) identified from Kaffirlime leaves seems to inhibit HSCs activation and reduce ECM deposition by the classical TGFβ1/Smad signaling pathway. Further proteomic analysis and molecular docking revealed a potential additional mode of action involving MAPK tyrosine kinase signaling proteins (22). It was found that exosomes of HepG2.2.15 cells play an important role in the activation, proliferation and fibrosis of LX2 cells. Moreover, KEGG pathway analysis highlights MAPK and other signaling pathways (23). Researchers have discovered that the gut microbiota metabolite 3-indolepropionic acid can activate human HSCs in a lab setting. The expression of fibrosis marker genes and the phosphorylation levels of p38 and JNK are increased by this activation. To confirm this discovery, inhibitors of p38 and JNK were used along with IPA to intervene in HSCs. As a result, the expression of fibrosis genes COL1A2, MMP9, and MMP2 decreased (24). In the study of the mechanism of SalB inhibiting HSCs autophagy and activation, researchers screened 11 protein sites with different expression. Among these, ERK, JNK, and p38 in the MAPK protein family were down-regulated (25). In addition,

SPA3014, a newly-synthesized selective histone deacetylase8, significantly downregulates MAPK-Smad2/3 and JAK2-STAT3 pathways to inhibit histone deacetylase8 and thus reduce the activation of HSCs to play an antifibrotic role (26). These evidences strongly suggest that the development of liver fibrosis is closely tied to the MAPK signaling pathway. Activation of this pathway accelerates HSCs activation, thereby exacerbating the disease. Conversely, inhibiting this pathway can effectively delay disease progression.

### 2.3 PI3K/AKT signal path

The phosphoinositide 3-kinase (PI3K) is a lipid kinase composed of heterodimers. It phosphorylates the phosphatidyl inositol lipid at the D-3 spot of the inositol ring in response to stimuli from growth factors and hormones. This process regulates cell growth, cycle, migration and survival (27). In mammals, the PI3K enzyme is encoded by different genes with distinct structural characteristics and specific phosphoinositol substrates. This categorization divides PI3K into three categories: I, II and III. Two subclasses, IA and IB, are the main divisions of Class I PI3K, which has been extensively studied. Subclass IA comprises the PI3Kα, PI3kβ, and PI3Kδ subtypes, while subclass IB only includes the PI3Kγ subtype (28). Class I PI3K is a complex composed of a regulatory subunit (p84, p85 or p101) and a catalytic subunit (p110α, β, γ, or δ). p110α and p110β are widely distributed in various tissues, whereas all leukocyte subtypes have high levels of p110γ and p110δ. The new study shows that p110δ levels are low but functionally correlated in white blood cells (29–31).

Cytokines, growth factors, and hormones act as ligands for G protein-coupled receptors (GPCRs) and receptor tyrosine kinases (RTKs) to activate them. Once activated, RTKs then act on the regulatory subunits of the three subtypes of class IA PI3K. In addition, GPCRs activate its downstream Gβγ subunit, which in turn activates PI3Kβ and PI3Kγ(32). The catalytic subunit p110 is activated by the regulatory subunits p85, p84 or p101. This activation results in the phosphorylation of the inositol ring at the 3′ position of phosphorylated phosphatidylinositol (4,5)-bisphosphate (PIP2) and leads to the formation of phosphatidylinositol (3,4,5)-trisphosphate (PIP3). The process can be restored to PIP2 by dephosphorylation of PIP3 under the action of Phosphatase and tensin homolog (PTEN) (33).

**FIGURE 3:**
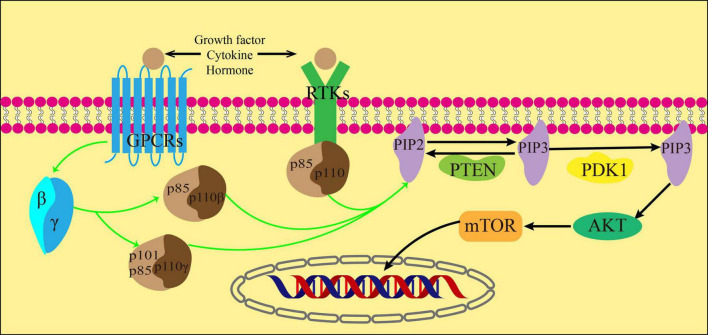
PI3K/AKT signal path.

Protein kinase B (AKT) is a serine/threonine kinase that connects the activation of growth factor receptors with the regulation of cell growth and metabolism. It plays a vital role in cell signaling (34). PIP3 recruits AKT and phosphoinositide-dependent kinase 1 (PDK1), a fellow Ph-domain kinase, and brings them together on the cell membrane’s surface. PDK1 then further causes AKT phosphorylation, which is decisive for AKT activation (35). After receiving biological information from PI3K, AKT transmits these signals to the downstream target mTOR through various cellular transcription factors. The activation of mTOR can inhibit autophagy by eliminating ubiquitin, which occurs through direct or indirect phosphorylation of AKT (36) (Figure 3).

In recent decades, many studies have found intricate connections between the dysregulation of the PI3K/AKT/mTOR signaling pathway and the occurrence and progression of various human diseases. Additionally, intervening in the dysregulation of this pathway has shown therapeutic potential for a variety of diseases (31). In previous studies on liver fibrosis, researchers found that the activation of the PI3K/AKT/mTOR pathway may be one of the mechanisms contributing to the onset and progression of hepatic fibrosis. The results of some studies using western blot analysis exhibited that the degree of P-PI3K, P-AKT, and P-mTOR proteins in the liver tissue of mice increased significantly after 6 weeks of intraperitoneal injection of CCl_4_, when contrasted with the control group. This confirmed that the PI3K/AKT/mTOR pathway was activated in the mouse model of liver fibrosis (37). To enhance comprehension of alcoholic liver fibrosis, researchers have investigated the PI3K/AKT/mTOR pathway. In contrast to CCl_4_-induced liver fibrosis, this study found that the degree of p-mTOR protein in the model band did not change significantly, and in some cases even decreased. However, the expressions of p-PI3K and p-AKT align with previous studies, suggesting that this signaling pathway is indeed activated in alcoholic liver fibrosis (38). After administering microRNA-101, interleukin-22 (IL-22) and exosomes derived from human adipose mesenchymal stem cells (hADMSCs-Exo), the expression levels of moderately phosphorylated molecules in the signaling pathways mentioned above were substantially reduced. This led to improved liver function and reduced liver parenchymal injury, as well as a decrease in the accumulation of ECM. These drugs also were found to inhibit the PI3K/AKT/mTOR pathway. As a result, they alleviated and even reversed liver fibrosis. This indicates that these drugs may have potential as a treatment for liver fibrosis (37–39).

In addition, the PI3K/AKT pathway is not limited to the PI3K/AKT/mTOR signal. Research on phosphoenolpyruvate carboxykinase 1 (PCK1) and metabolic-associated fatty liver disease (MAFLD) reveals that PCK1 deficiency increases GTP in cells, activating RhoA. This, in turn, triggers the PI3K/AKT pathway and raises PDGF-AA secretion, promoting HSC activation and increasing ECM deposition and fibrosis in the MAFLD model. Conversely, AKT/RhoA inhibitors have shown promise in delaying liver fibrosis progression, opening new possibilities for MAFLD treatment (40).

### 2.4 Wnt signaling pathway

The Wnt/beta (β)-catenin signaling pathway plays a crucial role in directing differentiation, proliferation, maintaining embryonic development and homeostasis. Additionally, it is engaged in the development of diverse human sickness (41). Research has demonstrated that trigger of the Wnt/β-catenin pathway is linked to fibrosis in various organ systems such as the liver, lung, kidney and skin. This association is particularly observed in the corresponding models. Inhibiting this pathway has substantial anti-fibrotic effects (42). Trigger of the Wnt channel can stimulate the propagation and activation of HSCs, leading to liver fibrosis by increasing extracellular matrix synthesis, epithelial-mesenchymal transition or interaction with other fibrotic mediators (43).

The Wnt signaling pathway consists of two different pathways, canonical and non-canonical pathways. The non-canonical pathway is participated in inflammation and lipid accumulation, whereas the canonical pathway is anti-inflammatory and antilipid proliferation and plays an antagonistic role against the non-canonical pathway (44). The classic Wnt/β-catenin pathway relies on β-catenin. β-catenin, a component of the cadherin complex found on the cell membrane, serves as a transmitter of intracellular signals in the Wnt channel. Changes in its function are linked to the onset of several liver diseases, such as liver fibrosis (45). It’s important to note that in normal liver tissue, this protein is found on the liver cell membrane. It attaches E-cadherin to the actin cytoskeleton and plays a role in intercellular adhesion. In damaged liver tissue, βcatenin is found in the cytoplasm as a downstream factor of the Wnt signaling pathway. Upon Wnt activation, β-catenin accumulates in the cytoplasm before translocating to the nucleus. Within the nucleus, it governs the transcription of specific genes through its interaction with the T-cell factor/lymphoid enhancer factor (TCF/LEF) family of transcription factors (46). During this path, β-catenin enlists the assistance of the cAMP response element-binding protein (CREB)-binding protein (CBP) or P300, which is akin to CBP, as coactivators in order to stimulate the transcription of various target genes (47).

**FIGURE 4:**
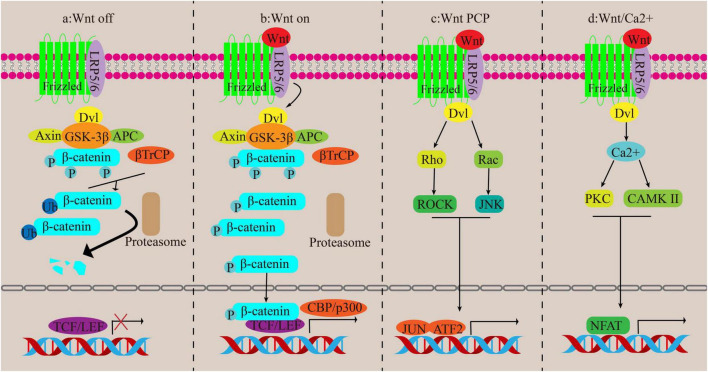
Canonical and non-canonical pathways of Wnt. **(a,b)** Canonical pathways. **(c,d)** Non-canonical pathways.

When the Wnt signaling pathway is turned off, β-catenin is kept at low levels in the cytoplasm and is controlled by a destabilizing complex consisting of axin, adenomatous polyposis coli (APC), glycogen synthase kinase 3β (GSK-3β) and casein kinase 1 alpha (CK1α). CK1 and GSK-3β phosphorylate ß-catenin and mark it for degradation through ubiquitination orchestrated by ß-catenin repeat sequence (ß-TrCP). Eventually, β-catenin levels become insufficient to activate the transcription process, leading to its degradation by the proteasome (48). Trigger of the canonical pathway occurs when the classical Wnt protein binds to the Frizzled transmembrane receptor and the low-density lipoprotein receptor-related protein 5/6 (LRP5/6). Axin relocates to LRP 5/6, leading to the inactivation of GSK-3ß, dissociation of the system, and dephosphorylation of β-catenin. Consequently, when β-catenin is not phosphorylated, it gathers in the cytoplasm and moves to the nucleus to kick-start the transcription of Wnt target genes (49) (Figures 4a, b).

The non-traditional Wnt signaling routes encompass the Wnt/Ca2 + channel and the PCP channel, both devoid of β-catenin participation. In the Wnt/PCP channel, the Wnt protein ties to its recipient and activates the disheveled (Dvl) gene, which then triggers the Rho/ROCK and Rac/JNK pathways, regulating cell functions. In the Wnt/Ca2 + channel, activation of the FZ receptor raises G proteins that actuate Dvl, leading to an increase in cytoplasmic Ca2 + concentration. This then triggers the activation of CAMK II and PKC, followed by the activation of nuclear factors involved in T cell-dependent transcription (43, 50) (Figures 4c, d).

OP-724, previously known as PRI-724, selectively inhibits CBP/beta-catenin. It inhibits the action of β-catenin with CBP through its active metabolite C-82. In mouse models of liver fibrosis, levels of cytokine and chemokine expression, as well as proteins associated with liver fibrosis, are elevated. Treatment with OP-724 can reduce this elevation and activate mitochondrial function, thereby improving liver function and showing anti-fibrotic properties (51, 52). Moreover, clinical studies have revealed that patients with cirrhosis of hepatitis B and C exhibit favorable tolerance to a dose of 280 mg/m^2^/4 h of PRI-724. After a 12-week treatment period, significant improvements in liver hardness, serum albumin levels and end-stage liver disease model scores were observed, with the patient’s Child-Pugh (CP) grade of liver function improving from class B to CP class A, showcasing the remarkable potential of OP-724 in clinical applications (53).

Indole alkaloids have been found to decrease organ fibrosis. Yohimbine hydrochloride (YHC), an indole alkaloid, has been studied for its impact on TGF-β induced Wnt/β-catenin ligand and receptor expression. When treated with TGF-β in comparison to the control group, the levels of classic Wnt components, Wnt frizzled receptors, and β-catenin were notably increased, while the addition of YHC significantly diminished the levels of these proteins in a dose-dependent manner with YHC (54). Similarly, 20(S)-protopanaxadiol (PPD), a major component of ginseng, weakened the Wnt/β-catenin pathway and depressed collagen deposition by decreasing TGF activity and increasing the levels of P-β-catenin and GSK-3β. Therefore, the degree of liver fibrosis was reduced in the therapy group (55). Furthermore, there is substantial evidence supporting the targeting of the Wnt pathway for liver fibrosis treatment. For example, CD73 regulates HSCs actuation and propagation through the Wnt/β-catenin pathway (56), gandouling alleviates liver fibrosis in Wilson’s disease through the Wnt-/β-catenin pathway (57), phillygenin restrains inflammation and the Wnt/β-catenin pathway improves liver fibrosis in mice treated with CCl4 (58), and so on.

### 2.5 NF-κB signaling pathway

The nuclear factor-κB (NF-κB) signaling pathway is a highly conserved evolutionary pathway involved in regulating of immunity, inflammatory response and cell function. The NF-κB family contains five subunits that bind to cellular DNA: p50, p52, cRel, p65 (also known as RelA) and RelB, encoded by *NF-*κ*B1*, *NF-*κ*B2*, *REL*, *RELA*, and *RELB*, respectively. The heterodimer p50/p65 stands out as the prevalent variant of NF-B and serves as a vital catalyst in the progression of liver cancer (59). Studies of the pathophysiological role of NF-κB in the liver have found that NF-κB is a central link between liver inflammation, fibrosis and hepatocellular carcinoma, suggesting that the NF-κB signaling pathway is a potential aim for preventing or even delay the advance of liver fibrosis (60). However, NF-κB has a dual role, and weakening the NF-κB signaling pathway can have beneficial effects while also having adverse effects on hepatocytes, especially when NF-κB is significantly inhibited (61).

**FIGURE 5:**
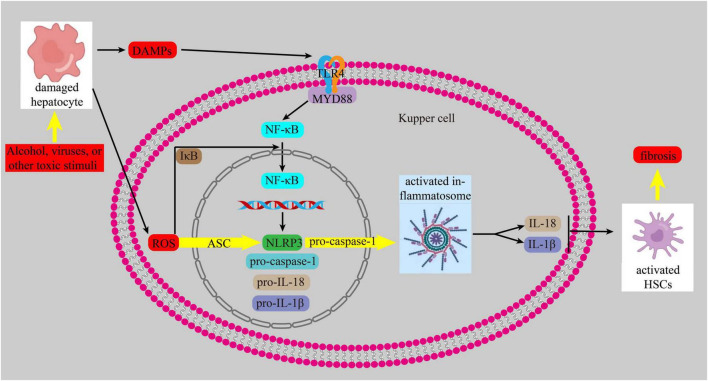
NF-κB signaling pathway.

Currently, it is increasingly recognized that inflammation and oxidative stress are intricately intertwined with the onset and advancement of liver fibrosis. When liver cells are exposed to harmful substances, they release reactive oxygen species (ROS) and damage-associated molecular patterns (DAMPs). These DAMPs activate Toll-like receptors (TLRs), tumor necrosis factor receptor, and IL receptor 1. The binding of DAMPs to their ligands triggers the TLR4/myeloid differentiation 88(MYD88) pathway, which then activates NF-κB. This leads to an inflammatory response by promoting the transcription of the NOD-, LRR- and pyrin domain-containing protein 3(NLRP3), similar to the nucleotide-binding oligomerization domain (NOD), procaspase-1, pro-IL-18, and pro-IL-1β. ROS also triggers the assembly of NLRP3 and the adapter apoptosis-related speckle, such as the apoptosis-associated speck-like protein containing a CARD (ASC), and recruits pro-caspase1, leading to the trigger of the inflammasome. Activated inflammasomes promote the growth of IL-1β and IL-18, which in turn activates HSCs, ultimately contributing to the development of liver fibrosis (62–64) (Figure 5).

A recent study demonstrated that ingestion of dibutyl phthalate (a plasticizer, considered a novel chemical pollutant) in mice resulted in NLRP3-mediated pyrodeath of hepatocytes via the NF-κB signaling pathway, activation of LX-2 cells, considerable ECM deposition and elevated hepatic fibrosis index (65). In contrast, fibroblast growth factor 21 (FGF21) inhibits HSC activation by blocking the NF-κB pathway, slowing the course of liver fibrosis (66).

In the past, antiviral drugs were agreed to have a hepatoprotective effect on rat liver fibrosis. Recently, it has been shown that the antiviral drugs Sofosbuvir and Velpatasvir exert their antifibrotic effects through the NF-κB pathway, and their antiviral activity does not appear to be involved in this process. Compared to the model group induced by CTC alone, Sofosbuvir and Velpatasvir alone or in combination could reduce the expression of TNF-α, thus downregulating the NF-κB pathway, and minimize the number of activated HSCs. Reducing or degrading the ECM produced showed an antifibrotic effect (67). In addition, there is increasing evidence for the antifibrotic effect of polysaccharides. For example, Gardenia jasminoides can reduce liver damage and fight fibrosis, but the specific component that plays a role is unclear. Based on its structure and biological activity in plant cells, pectin is an integral part of polysaccharides (68). Therefore, some scholars assumed that the polysaccharides in Gardenia jasminoides had this effect and extracted the homogeneous polysaccharide GJE0.2-2 rich in pectin

from Gardenia Jasminoides. GJE0.2-2 substantially suppressed HSCs actuation in mice with CCl_4_-induced hepatic fibrosis and reduced collagen deposition. When TGF-β1 is added to LX-2 cells, GJE0.2-2 can directly tie to TLR4 and dephosphorylate the downriver inhibitory protein IκB, resulting in NF-κB binding of NF-B to IκB and retention in the cytoplasm, ultimately leading to the inhibition of NF-κB nuclear translocation in LX-2 cells. Therefore, blocking the activation of HSCs by blocking the NF-κB pathway, indicating the beneficial effect of GJE0.2-2 on the settlement of liver fibrosis. In addition, GJE0.2-2 increased TGF-β induced ROS (69). There are many examples of reducing the degree of liver fibrosis by inhibiting NF-κB signaling pathway, such as salvianolic acid B (70), empagliflozin (71), etc.

### 2.6 AMPK signaling pathway

Adenosine 5′-monophosphate-activated protein kinase (AMPK) is a heterotrimer made up of two regulatory subunits (β and γ) and a catalytic component (α). As a part of the Serine/threonine kinase family, it is located in varieties of organs (heart, brain, lung, liver, kidney, etc.) (72). AMPK is a key player in the regulation of energy balance. It does this by limiting anabolic pathways which lower ATP consumption, and stimulating catabolic pathways which raise ATP synthesis (73). There is growing evidence that AMPK can prevent fibrosis of the heart (74), liver (75), lungs (76), etc.

In mammals, the two main AMPK-activated cyclic phosphorylated kinases are Ca2 + /calmodulin-dependent protein kinase β (CaMKK2) and tumor suppressor liver kinase B1 (LKB-1) in the Strad-MO25 complex (77). AMPK’s phosphorylation and activation are influenced by LKB1, an AMPK upstream kinase. In the liver, deletion of the LKB1 gene results in phosphorylated AMPK being downregulated and AMPK signaling events not being sensed (78). When intracellular Ca2 + concentration is elevated, CaMKK2 directly phosphorylates AMPK on Thr172 to increase its activity (79).

In a CCL4-induced mouse liver fibrosis model, water extract of earthworms (WEE) increased liver LKB1, AMPK, and GSK3β phosphorylation levels. Similarly, Comparable outcomes were observed in LX-2 cells. After activation of LKB1/AMPK/GSK3β, Nrf2 downstream of LKB1 was induced to enter the liver nucleus and the antioxidant factor level of Nrf2 downstream increased. In AML-12 hepatocytes and LX-2 HSCs, treatment with WEE increased intracellular Nrf2 levels, facilitated its movement into the nucleus and hindered the accumulation of ROS induced by TGF-β1. After knockout of LKB1, the effect of WEE on the AMPK/GSK3β/Nrf2 cascade was also eradicated, and its defensive effect anti-TGF-β1 was eliminated (80). Other researchers evaluated the expression of autophagy-related proteins and AMPK pathway-related proteins in mouse liver tissue, verified that ACE2 overexpression can regulate HSCs autophagy through the AMPK/mTOR pathway, thus alleviating liver fibrosis and liver sinus remodeling by inhibiting HSCs activation and promoting apoptosis (81). In contrast, AMPK pathway agonists can significantly reduce the expression levels of fibrosis marks such as a-SMA, TGF-β and collagen1 in both *in vivo* and *in vitro* researches, thus reducing the degree of liver fibrosis (82). In addition, theaflavine was found to up-regulate AMPK activity in HSC LX-2 and mouse livers. Phosphorylation levels of GSK3β, a typical substrate of AMPK, were correspondingly elevated. On the contrary, AMPK inhibitors significantly promoted theaflavine-induced HSCs activation *in vitro* (82). In clinical studies, AMPK phosphorylation is reduced in the liver of hepatitis C patients, while the level of fibrosis markers is significantly upregulated, and activation of the AMPK pathway can eliminate fibrosis caused by hepatitis C infection (83).

## 3 The internal crosstalk among the above multiple signal pathways

As mentioned earlier, activation of HSCs is a key step in the development of liver fibrosis. There are multiple signaling pathways involved in the activation of HSCs. However, these signaling pathways are not solely involved in this process.

First, these signaling pathways can be activated simultaneously or sequentially through their respective receptors in response to external stimuli, thus playing a combined role. LPS is a specific ligand for TLR4 on HSCs membranes, it can trigger more than one of signaling pathways to activate HSCs to promote liver fibrosis, including NF-κB and MAPK (84). Instead, an intervention could block multiple signaling pathways simultaneously, thereby preventing HSCs activation and delaying liver fibrosis. Schisantherin A significantly inhibited the proliferation and activation of HSCs by inhibiting the expression of proteins associated with MAPK and NF-κB signaling pathways (85). Taxifolin also inhibits HSCs activation and ECM production by regulating the PI3K/AKT/mTOR and TGF-β1/Smads (86). Helenalin inhibits HSCs activation by inhibiting Mir-200A-mediated PI3K/Akt and NF-κB pathways, and is a potential drug for the treatment of liver fibrosis (87). In liver fibrosis patients with diabetic, blunting p38 MAPKα and ERK1/2 activities by empagliflozin enhances the antifibrotic effect of metformin and augments its AMPK-induced NF-κB inactivation. The metformin/empagliflozin combined therapy could be promising in preventing hepatic inflammation and fibrosis via exhibiting complementary effects (88).

What’s more, there are interactions between these signaling pathways. Activation of one pathway can activate or enhance the action of another pathway. In addition to activating the Smad pathway, TGF-β can also activate non-Smad signaling pathways, such as PI3K/AKT, MAPK, NF-κB, and others. These pathways can regulate the classical Smad pathway and affect TGF-β-mediated biological responses (89–91). Salvia miltiorrhiza and Radix astragali can regulate the expression of CyclinD1, a key factor in Wnt/β-catenin signaling pathway. The expression of CyclinD1 is closely related to the activation of hepatic stellate cells and liver regeneration. Interestingly, the regulatory effect of the herbal extract on β-catenin was much less pronounced than that on the expression of CyclinD1. This suggests that other signaling pathways are involved in regulating the expression of CyclinD1, such as interleukin-8, MAPK, and PI3K (92, 93). In addition, NF-κB and MAPK signal transduction have interactions, and MAPK activation can lead to the phosphorylation of IκB/IκB and the movement of NF-κB p65 to the nucleus (94, 95). Tianhuang formula inhibits the activation of HSCs by significantly inhibiting the activation of p38 MAPK and NF-κB p65, thereby inhibiting liver fibrosis (96).

## 4 Potential therapeutic effects of various signaling pathways in hepatic fibrosis

So far, the development of anti-fibrosis therapeutics represents an unsolved area with great potential. Of course, previous studies have also explored a large number of treatment methods targeting the above signaling pathways to prevent the activation of HSCs, thereby alleviating liver fibrosis. They include: drugs (Table 1), bioactive molecules in natural products (Table 2), and small molecule compounds (Table 3).

**TABLE 1:** Potential therapeutic drugs targeting various signaling pathways.

Name	Classification	Intervention mechanism	References
Praziquantel	A schistosomicide	Praziquantel inhibits activation of HSCs via Smad7 up-regulation.	(97)
Saroglitazar	A dual PPAR-α/γ agonist	Saroglitazar inhibits the TGF-β/Smad signal path.	(98)
Vildagliptin (VLD)	A DPP-4 inhibitor	VLD attenuated hepatic fibrosis by MAPK/ERK1/2 (MEK1/2), ERK1/2, p38α, NF-κB and Smad2/3.	(94)
Carvedilol	A non-selective βblocker	Carvedilol increased SMAD7, attenuated the pro-fibrogenic marker TGF-β1 and the inflammatory markers (p-38 MAPK and p-S536-NF-κB p65).	(99)
Tenofovir disoproxil fumarate (TDF)	Antiviral drugs	TDF directly ameliorates liver fibrosis by downregulating the PI3K/Akt/mTOR signaling pathway, which results in the apoptosis of activated HSCs.	(100)
Idelalisib	Highly selective oral phosphatidylinosit ol 3-kinase δ (PI3K-δ) inhibitor	Idelalisib promote the apoptosis of activated HSC cells by inhibiting the PI3K/AKT/FOXO3 signal pathway.	(101)
Doxazosin	A traditional α1adrenergic receptor (α1AR) antagonist	Doxazosin activates the PI3K/Akt/mTOR signaling pathway, thereby inhibits autophagy and induce the apoptosis of activated HSCs.	(102)
Yu Gan Long (YGL)	A Chinese traditional herbal formula	YGL reduces inflammation cytokines production, and suppresses PI3K/AKT signaling pathways.	(103)
Octreotide	Somatostatin analogue	Octreotide inhibit HSCs activation and ECM synthesis via the suppression of the PI3K/AKT signaling pathway.	(104)
Forsythiaside A (FA)	An effective component isolated from traditional Chinese medicine Forsythi a suspensa	FA inhibited PI3K/Akt pathway to suppress HSCs proliferation.	(105)
BI 113823	Inhibitor of k inin B1 receptors	BI 113823 inhibited TGF-β and B1R agonist-stimulated human-HSC activation, contraction, proliferation, migration and fibrosis protein expression, and inhibited activation of PI3K/Akt signal pathway.	(106)
OP-72	A selective CBP/βcatenin inhibitor	The inhibition of CBP/β-catenin signaling by administration of OP-724 ameliorated fibrosis in the liver.	(51)
Dasatinib	A secondgeneration oral multitarget inhibitor of many tyrosine kinases	Treatment with dasatinib downregulated miRNA-17 expression, leading to the restoration of WiF-1 and smad-7 which cause the inhibition of both Wnt/β-catenin and TGF-β/smads signal. In addition, it upregulated miRNA-378 leading to the decrease of Wnt-10 which contributes to the suppression of activated HSCs.	(107)
Fenofibrate	PPARα agonist	Fenofibrate reduced hepatic iron accumulation and prevented ironinduced downregulation of liver Sirt3 and active β-catenin, mitigating the progression of fibrosis.	(108)
Niclosamide	An oral anthelmintic drug of tapeworm infection	Niclosamide also significantly reduced NOTCH pathway, Wnt pathway, and fibrosis,α-SMA and collagen deposition.	(109)
Vitamin D and puerarin	Combination	The combined use is able to silence the Wnt1/β-catenin pathway, suppress the activation of hepatic stellate cells, and reduce the secretion of collagen fibers	(110)
Octreotide	Somatostatin	Octreotide attenuate liver fibrosis by inhibiting Wnt/β-catenin signaling pathway.	(111)
ICG-001	β-catenin/CBP inhibitor	ICG-001 suppres stromal CXCL12 suggests a potential therapeutic approach targeting activated HSCs in liver fibrosis.	(112)
Diosmin (Dios)	A natural citrus flavone	Dios treatment repressed the miR-175p activated Wnt–β-catenin signaling induced by IRR.	(113)
Elafibranor (EFN)	A dual PPARα/PPARδ agonist	EFN reduced ALD-related fibrosis by suppressing LPS/TLR4/NF-κBmediated inflammatory responses by restoring intestinal barrier function.	(114)
Tianhuang formula (THF)	A drug combination consisting of 2 traditional Chinese herbs	THF ameliorated liver injury, inflammation and fibrotic process by inhibiting CCL2-CCR2 axis and its downstream MAPK/NF-κB signaling pathway.	(96)
Praziquantel	A schistosomicide	Praziquantel inhibits activation of HSCs via Smad7 up-regulation.	(97)
Empagliflozin (EMPA)	A novel sodium glucose cotransporter	EMPA could exert and potentiate its anti-inflammatory and anti-fibrotic effects using NASH rat model via	(71)
	Inhibitor	targeting hepatic NF-κB/SOX 9/OPN axis and OCN.	
J2H-1702	11βHSD1 inhibitor	After treatment of J2H-1702, expression of genes related to NF-κB activation (TLR7, ITGB3, and TWIST), which generally promote inflammation, fibrosis, and hepatocarcinogenesis in non-parenchymal cells were downregulated.	(115)
Edaravone (EDA)	A strong novel free radical scavenger	EDA inhibited of NF-κB signal pathway and reactive oxygen species (ROS) production in macrophages. Moreover, EDA treatment indirectly suppressed the activation of HSCs by decreasing the IL-1β secretion of macrophages.	(116)
TJ- M2010-5	Myeloid differentiation factor-88 (MyD88) MyD88 inhibitor	TJ-M2010-5 upregulated the expression of bone morphogenetic protein and membrane-bound inhibitor in LX2 cells by blocking the activation of MyD88/NF-κB, thereby inhibiting the phosphorylation of Smad2/3 and the expression of collagen I.	(117)
Sitagliptin	An oral hypoglycemic agent	Sitagliptin treatment resulted in downregulation in the immunoexpression of NF-κB and its downstream cytokine, TNF-α.	(118)
Dapagliflozin (DAPA)	A sodium-glucose cotransporter-2 (SGLT2) inhibitor	DAPA suppressed endothelial cell inflammation and apoptosis induced by high glucose via activating the AMPK/Sirt-1 pathway and elevating PCG1α levels.	(119)
Metformin	Hypoglycemic	The inhibitory effects of metformin on activated HSCs were mediated by inhibiting the Akt/mammalian target of rapamycin (mTOR) and extracellular signal-regulated kinase (ERK) pathways via the activation of adenosine monophosphate-activated protein kinase (AMPK).	(120)

**TABLE 2:** Bioactive molecules in natural products targeting various signaling pathways.

Name	Origin	Intervention mechanism	References
Andrographolide (Andro)	The aerial parts of plants of the genus Andrographis	Inhibition of the TLR4/NF-κB signaling pathway by educed the phosphorylation and nuclear translocation of NF-κB p50; downregulated the TGF-β1/Smad2 pathway independent of Smad7.	(121)
Naringin	A key component of GSG	Naringin deactivated HSCs through the impediment of the Smad signaling cascade.	(122)
Presegetane diterpenoid	The aerial parts of Euphorbia sieboldiana Morr	Presegetane diterpenoid inhibit TGFβ/Smad signaling pathway via its potential target was TGF-β type I receptor.	(123)
Ligustri Lucidi Fructus (LLF)	The fruit of Ligustrum lu cidum Ait	LLF down-regulated TGF-β/Smad signaling pathway on the protein expression of Smad2/3 phosphorylationdecreasing.	(124)
Cannabidiol (CBD)	An abundant non-psychoactive component in the cannabis plant	Inhibition the expression of p-p38 MAPK.	(125)
Carvacrol	Aromatic plants	Carvacrol inhibit the expression of TRPM7 and inhibit the proliferation and activation of HSCs to alleviate liver fibrosis by modulating MAPK signaling pathway.	(126)
Honokiol	Magnolia species	inhibited TGF-β/SMAD/MAPK signaling pathways.	(127)
Lycorine	Amaryllidaceae genus	Lycorine inhibited PI3K/AKT phosphorylation in a dose-dependent manner.	(128)
Taxifolin	Pine plants	Taxifolin inhibited the activation of hepatic stellate cells and the production of extracellular matrix (ECM) by regulating PI3K/AKT/mTOR and TGF-β1/Smads pathways.	(86)
Asiatic acid (AA)	The triterpenoid components of Centella asiatica	AA inhibited hepatic stellate cell activation and extra cellular matrix (ECM) synthesis by regulating the PI3K/AKT/mTOR signaling pathway.	(129)
Quercetin (3,3,4,5,7 pentahydroxyflavone, QE)	A flavonoid present in fruits and vegetables	Quercetin ttenuated liver damage by suppressing the TGF-β1/Smads signaling pathway and activating the PI3K/Akt signaling pathway to inhibit autophagy.	(130)
CCM111	Antrodia cinnamomea	CCM111 prevents hepatic fibrosis via cooperative inhibition of TGF-β, Wnt and STAT3 signaling pathways.	(131)
Morin	Moraceae plants	Morin by acting on Hippo/Yap and TGFβ1/Smad pathways, ameliorated experimental liver fibrosis through prevented HSC activation.	(132)
Arbutin	Bearberry of azalea family	Arbutin ameliorates liver inflammation and fibrosis in mice by inhibiting hepatic stellate cell activation via reducing macrophage recruitment and infiltration and suppressing activation of the Akt/NF-κB and Smad signaling pathways.	(133)
Mangiferin	Mango	Mangiferin alleviate liver fibrosis by reduced collagen accumulation and HSCs activation, inhibited the p-IκB and p-p65 protein levels.	(134)
6-Shogaol	Rhizome of Zingiber officinale Roscoe	6-Shogaol can prevent CCl4-induced liver fibrosis by suppressing inflammatory response through the NF-κB pathway.	(135)
Baicalin (BA)	Radix Scutellariae	BA presented a possible anti-fibrotic effect by inhibiting inflammation provoked by NF- κB/IL-6 and NF-κB/NLRP3 inflammasome/IL-1β. Finally.	(136)
Resveratrol (RSV)	Red wine and peanuts	RSV significantly decreased transforming growth factor-β synthesis and inflammatory factor expression and reduced the inflammation of hepatic stellate cells by inhibiting the NF-κB pathway *in vivo* and *in vitro*.	(137)
Spore powder of Antrodia camphorata (SP)	Antrodia camphorata	In summary, SP has an ameliorative effect on hepatic fibrosis, probably by inhibiting the activation of hepatic stellate cells, reducing the synthesis of extracellular matrix by down-regulated the protein expression of toll like receptor 4 (TLR4) and nuclear factor-K b (NF-κB) p65.	(138)
Forsythiae Fructuse water extract (FSE)	Forsythiae Fructuse	FSE can inhibit the expression of inflammatory factors and fibrotic cytokines, reduce liver injury, and inhibit the development of liver fibrosis through TLR4/MyD88/NF-κB and TGF-β/smads signaling pathways.	(139)
Arctigenin (ATG)	The seeds of burdock	ATG can activate AMPK/PPARγ pathway to restore the activated hepatic stellate cell to quiescence thereby improving liver fibrosis.	(140)
Cordycepin	Fungus Cordyceps militaris	Cordycepin promoted AMPK phosphorylation and activated the AMPK downstream pathway to prevent hepatic steatosis, inflammation, and fibrosis.	(141)

**TABLE 3:** Small molecule compounds targeting various signaling pathways.

Drug	Classification	Intervention mechanism	References
Krüppel-like factor 10 (KLF10)	A zinc fingercontaining transcription factor	KLF10 suppresses TGF-βinduced HSC activation by targeting the expression of activating transcription factor 3.	(4)
J-1063	The synthesis of a pyrazole derivative	J-1063 exerted anti-fibrotic activity on TGF-β-induced hepatic stellate cells activation by inhibiting TGF-βR1 (ALK5).	(142)
N-n-Butyl haloperidol iodide (F2)	A novel compound derived from halopendol	F_2_ suppressed the binding of c-Jun to the TGFBR2 promoter to restrain TGF-β signaling and inhibit α-SMA and collagen I upregulation.	(143)
Bone morphogenetic protein 7 (BMP7)	A member of the TGF-β family	BMP7 inhibited TGF-β1-induced activation, migration, and proliferation of HSCs via upregulation of pSmad1/5/8 and downregulation of phosphorylation of Smad3 and p38MAPK.	(144)
miR-130a-3p	microRNA	miR-130a-3p inhibit the activation and proliferation of HSCs but also induce the apoptosis of HSCs by inhibiting the expressions of MAPK and TGFBR.	(145)
lncRNA ANXA2P2 (mouse Anxa6)	lncRNA	LncRNA ANXA2P2 dynamically modulates the phosphorylation of PI3K and Akt.	(146)
miR-200a	microRNA	miR-200a negatively regulate HSC activation, proliferation, and migration by inhibiting PI3K/Akt and NF-κB pathways.	(87)
Thymosin β4 (Tβ4)	G-actin chelating peptide found	Tβ4 inhibits hepatic apoptosis, and fibrosis induced by suppressing the PI3K-AKT-NFκB pathway.	(147)
miR-101	microRNA	miR-101 exerts antifibrotic effect by downregulating the PI3K/Akt/mTOR signaling pathway.	(37)
lncRNA GAS5	lncRNA	lncRNA GAS5 restrains hepatic fibrosis by targeting miR-23a through the PTEN/PI3K/Akt signaling pathway.	(148)
Maltol	A food flavoring dditive	Maltol alleviated experimental liver fibrosis by suppressing the activation of HSCs and inducing apoptosis of activated HSCs through TGF-β1-mediated PI3K/Akt signaling pathway.	(149)
Phosphatase and Tensin Homolog Deleted on Chromosome Ten (PTEN)	A dual specificity protein and lipid phosphatase	PTEN blocked serum-induced phosphorylation of Akt, p70^S6K^, and Erk in HSCs.	(150)
Adiponectin	A 30 kDa adipocytokine	Adiponectin-induced upregulation of miR-29b can suppress DNMT3B transcription in LX-2 cells, thus resulting in reduced methylation of PTEN CpG islands and ultimately suppressing the PI3K/AKT pathway.	(151)
CD147	A transmembrane glycoprotein	CD147 regulates CXCL1(promoted HSCs activation) release in HSCs by PI3K/AKT signaling.	(152)
Doublecortin domain containing 2 (DCDC2)	protein; a member of the DCX family	DCDC2 inhibited TGF-β1- induced HSC activation partly through the Wnt/β-catenin signaling pathway.	(153)
Compound 38	The bromodomain and extra- terminal (BET) family of chromatin proteins	Compound 38 inhibited the Wnt/β-catenin and transforming growth factor-beta/SMAD signaling pathways to abolish the activation of HSCs.	(154)
Brahma-related gene 1 (Brg1)	An enzymatic subunit of the switch/sucrose non-fermentable complex	Brg1 binds to the β-catenin/TCF4 transcription complex.	(155)
miR-16	microRNA	miR-16 targets a set of signaling pathways essential for myofibroblasts, such as Wnt and TGF-β, thereby inducing the resolution of liver fibrosis.	(156)
PLK1 (polo-like kinase 1)	Polo-like kinase family	The Wnt/β-catenin signaling pathway may be essential for PLK1-mediated HSCs activation	(157)
Krüppel-like factor 10 (KLF10)	A zinc fingercontaining transcription factor	KLF10 suppresses TGF-βinduced HSC activation by targeting the expression of activating transcription factor 3.	(4)
Heme oxygenase-1 (HO-1)	Heat shock Protein 32	HO-1 inhibited the activation of canonical and non-canonical Wnt signaling pathways in NASHrelated liver fibrosis.	(158)
human bone mesenchymal stem cells- derived exosomes (hBM- MSCs-Ex)	Exosomes (30– 100 nm)	hBM-MSCs-Ex treatment could ameliorate CCl_4_-induced liver fibrosis via inhibition of HSC activation through the Wnt/βcatenin pathway.	(159)
Notum	A newly discovered inhibitor to Wnt proteins	Notum inhibited HBV-induced liver fibrosis through downregulating Wnt 5a mediated non-canonical pathways.	(160)
YAP (Yesassociated protein)	A transcriptional coactivator	YAP attenuated Wnt/β-catenin pathway activity in activated HSC-T6 cells.	(161)
ZM600	Sophoridine αaryl propionamide derivative	ZM600 has a protective effect on liver fibrosis by inhibited the activation of NF-κB, PI-3K/AKT, and TGF-β/Smads signaling pathways.	(162)
Lactoferrin (LF)	Iron-binding glycoprotein	LF might act as a chemopreventive agent to prevent hepatic injury, inflammation, and fibrosis in NASH via NF-κB inactivation.	(163)
miR-124	microRNA	miR-124 repressed the inflammation cytokines secretion of TNF-α-induce HSCs by inhibiting activation of the NF-κB signaling pathway.	(164)
Hesperetin derivative (HD16)	A monomer compound derived from hesperitin	HD-16 attenuated CCl_4_-induced liver inflammation and fibrosis by activating the AMPK/SIRT3 pathway.	(165)
Fibronectin type III domain- containing protein 5 (FNDC5)	A novel myokine	FNDC5 plays beneficial roles in attenuating liver fibrosis via AMPK phosphorylation-mediated inhibition of HSCs activation.	(166)

## 5 Conclusion and outlook

Chronic liver disease of multiple etiologies, such as toxic injury, viral infection, autoimmune diseases, metabolic disorders and genetic ailments, can develop liver fibrosis. Liver fibrosis is regarded an intermediate stage, and effective measures can delay or even reverse its process. Otherwise, it progresses to cirrhosis and the end of liver diseases (96). HSCs activation in liver fibrosis has been shown to is directly connected to the progression of liver fibrosis. Under the action of pro-inflammatory factors, HSCs activation is promoted through various signaling pathways mentioned earlier. The activated HSCs then proliferate, become more contractile and transform into fibroblast-like fibroblasts that secrete a large amount of ECM. It showed fibrosis *in vitro*. *In vivo*, it leads to liver tissue remodeling and angiogenesis, and then promotes the progress of liver fibrosis.

The evidence summarized here clearly shows that HSCs activation involves activation of multiple signaling pathways. However, these signaling pathways do not play a role alone, but a variety of signaling pathways often work together to participate in the activation of HSCs. For example, a polysaccharide from the roots of Codonopsis pilosula can promote the activation of HSCs *in vivo* and *in vitro* through the combined action of TGF-β1/Smad3 and TLR4/NF-κB signaling pathways, eventually promote liver fibrosis (167). In addition, there are also many non-coding RNAs (including long non-coding RNAs, microRNAs and circular RNAs) through the TGFβ pathway, Wnt/βcatenin pathway,PI3K/AKT pathway and other pathways are engaged in the liver fibrosis (168).

Since the activation of HSCs is crucial in driving the advancement of liver fibrosis, we thought of two ways to delay or even reverse liver fibrosis. On the one hand, inactivation of activated HSCs is one way to induce senescence. However, the persistence of senescent cells can interact with inflammatory cells and reshape the microenvironment to further induce aging-related dysfunction (169). Therefore, this approach needs to be further studied. As mentioned earlier, HSCs activation is inhibited by blocking the signaling pathways mentioned above. In the past, we have conducted extensive studies on the molecular mechanisms of HSCs activation in liver fibrosis. We have also tabulated some interventions that have been found in recent years to prevent liver fibrosis. They include drugs, bioactive molecules in natural products and small molecule compounds that target the above signaling pathways to prevent HSCs activation. However, these methods are still in the basic research stage and have little application in clinical practice. Furthermore, there are still few studies on the potential synergistic mechanisms that may exist in various pathways of HSCs activation. Therefore, there is had no choice but to explore new potential targets by substantial amounts of preclinical and clinical trials, and more attention should be paid to the combined action of multiple signaling pathways that activate HSCs in order to solve the urgent need of liver fibrosis treatment.
